# Toxicity profile and early clinical outcome for advanced head and neck cancer patients treated with simultaneous integrated boost and volumetric modulated arc therapy

**DOI:** 10.1186/s13014-015-0535-0

**Published:** 2015-11-06

**Authors:** Ciro Franzese, Antonella Fogliata, Elena Clerici, Davide Franceschini, Elisa Villa, Giuseppe D’Agostino, Piera Navarria, Pietro Mancosu, Stefano Tomatis, Luca Cozzi, Marta Scorsetti

**Affiliations:** Radiotherapy and Radiosurgery Department, Humanitas Research Hospital, Milan, Rozzano 20089 Italy

**Keywords:** Advanced head and neck cancer, VMAT RapidArc, Simultaneous integrated boost SIB, Toxicity profile

## Abstract

**Background:**

Shortening the overall treatment time without increasing acute reactions is one of the major aims in radiotherapy for head and neck cancer (HNC). Volumetric modulated arc therapy (VMAT) with Simultaneous Integrated Boost (SIB) showed improvements in outcome and pattern of toxicity. Patients with stage III-IV HNC treated with VMAT-SIB have been analysed, and doses were correlated to limiting structures and toxicity.

**Methods:**

One hundred two patients treated from December 2008 to August 2014 were analysed. Patients were treated with VMAT (RapidArc) and SIB in 33 fractions for a total dose of 69.96 and 54.45Gy, respectively. For organs at risk, D_1/3 V_, D_1/2 V_, D_2/3 V_, the mean dose, V_D_ with *D* = 10,20,30,40,50,70 Gy were analysed. For targets, D_98%_, D_2%_, and V_95%_, V_107%_, conformity and homogeneity indexes were calculated. Toxicity was graded according to CTCAE3.

**Results:**

Oral cavity V_30Gy_, V_40Gy_, and V_70Gy_, were found correlated with mucosal toxicity grading. Concerning salivary glands, significant was only D_2/3V_ for one of the two parotids. Almost all analysed parameters of the inferior constrictor muscle were significant while no correlations were found for middle and superior constrictors. With median follow-up of 19 months, Overall Survival (OS) at 3 and 5 years was 83 % ± 4 % and 73 % ± 10 %. Mean OS was 51 ± 3 months. Disease Free Survival (DFS) at 3 and 5 years was 71 % ± 7 %, and 34 % ± 16 %. Mean DFS was 43 ± 3 months.

**Conclusions:**

RapidArc technology and SIB with 1.65 and 2.12Gy/fraction for 33 fractions showed a good toxicity profile and encouraging trend for OS and DFS for patients with stage III-IV HNC.

## Background

Altered fractionation started to be explored in the 80-ies trying to improve head and neck cancer (HNC) treatment outcome [1]. Mainly two classes of fractionation schedules showed to be promising: the acceleration and the hyper-fractionation. The first one aims to reduce the proliferation of the tumour by shortening the total duration of the radiotherapy treatment, increasing the number of fractions/week, and/or the fractions/day. With the hyper-fractionation regimen (based on the high α/β ratio of the HNC, as the early responding tissues [2]), higher total doses are delivered with smaller dose/fraction.

Accelerating the treatment by one week without dose reduction, as well as using hyper-fractionation, an increase in loco-regional control of intermediate to advanced carcinomas was observed without increasing the late toxicity. An improvement in patient survival can be achieved also with the combination of radiotherapy and concurrent chemotherapy in patients with advanced tumours, at expenses of an increased morbidity. However, when the overall duration of the treatment is greatly reduced, the acute reactions become not acceptable, and the total dose or the dose/fraction has to be decreased.

The choice of the fractionation scheme in HNC patients is a delicate problem, as many factors are involved. The key point would be to shorten overall treatment time without increasing acute reactions.

Also pragmatic factors, as the workload, the costs, the logistic convenience of a centre to treat patients more than once a day, or during weekend, has to be taken into account when choosing the fractionation scheme for HNC patients.

With the advances in linear accelerator technologies and the advent of intensity modulation, the treatment deliveries know today a significant improvement. Organs at risk (OAR) are possible to be spared to dose level below tolerance values while keeping high and homogenous doses delivered to the target volume. Improvements in diagnostic imaging lead to more accurate tumour delineation. All such new advances need today to be included in the biological knowledge about the altered fractionation in order to understand which could be the proper fractionation schedule, total dose and dose/fraction that maximize the tumour cells killing while minimizing the acute and late toxicities.

An option available with the new technologies is the Simultaneous Integrated Boost (SIB) approach that, even with different associated biological mechanisms due to the different treatment timing, could be considered as an alternative to the hyper-fractionation with the concomitant boost which showed some improvements.

Many dosimetric studies proved that volumetric modulated arc therapy (VMAT) could deliver very conformal treatments highly sparing most of the surrounding critical structures. Even HNC treatments were studied to explore the dosimetric benefits from VMAT [3–5].

In the current literature, very few reports about HNC patients treated with VMAT technology and SIB fractionation are published. Until now, only two studies explored the correlation between dosimetric data and acute toxicity [6, 7], all with a few number of patients. To notice are also the different approaches of the three studies in terms of fractionation, making it difficult for a direct comparison. Scorsetti et al. [6] reported about the initial clinical experience on 45 patients treated with VMAT for different HN sites and stages; Smet et al. [7] compared clinical and dosimetric results of 78 patients treated with IMRT and 79 patients treated with VMAT. Another study, Doornaert et al. [8], presented dosimetric results on 35 HN patients.

Aim of the present study was to analyse the outcome data for a group of 102 patients presenting stage III and IV HNC and treated with VMAT based SIB with a common fractionation scheme. In detail, the study focused on the assessment of possible correlation between the planned dose distributions to the main dose limiting structures and the observed levels of toxicity like mucositis, xerostomia and dysphagia at both acute and late levels.

## Methods

### Patient selection

This study consisted in a retrospective dosimetric and clinical evaluation of a cohort of 102 patients treated in our department from December 2008 to August 2014. All patients were treated in agreement with the Helsinki declaration. The retrospective analysis of treatment charts have been approved by the Humanitas Cancer Center ethical committee. All patients presented advanced HNC, stage III and IV according to the American Joint Committee on Cancer staging system.

Induction chemotherapy treatment was administered to 54 % of the cases, while concomitant chemotherapy was administered to 91 % of the patient cohort.

### Dose prescription and treatment

All patients were treated with VMAT in its RapidArc form, with SIB in 33 fractions of 2.12Gy to the boost volume, and of 1.65Gy to the elective volume, for a total dose of 69.96Gy and 54.45Gy, respectively. The 69.96Gy were prescribed to the mean PTV (planning target volume).

The gross tumour volume (GTV) was delineated on CT imaging for all patients, MR imaging was co-registered in 63 cases and PET imaging in 64 cases. The clinical target volume (CTV) for boost encompassed the GTV with an additional 1 cm margin for both the tumour and nodal disease, correcting for anatomical boundaries; the elective CTV included also the elective nodes according to internationally accepted guidelines [9]. An isotropic 5 mm margin was then added to CTVs to obtain the PTVs. PTVs were cropped 4 mm inside the body contour.

RapidArc treatments used 6MV beams from a TrueBeam, a Clinac DHX, or a Unique linear accelerator (Varian Medical Systems, Palo Alto, USA). All treatment units were equipped with a Millennium 120-MLC. Treatment plans were optimized for two to four full arcs with different collimator angles according to the anatomy complexity. The Progressive Resolution Optimizer engine was used for inverse planning and doses were estimated with AAA from the Varian Eclipse treatment planning system (versions 8.5 to 11).

### Dose planning objectives and dose evaluation

For PTV coverage, the 95 % of the prescribed dose was requested to cover 95 % of the target volume (V_95%_ > 95 %), for both boost and elective volumes. The near-to-maximum dose to the boost (D_2%_) was constrained below 105 % of the prescription for the boost volume, while it has to be minimized for the difference volume between elective and boost.

The following OARs were considered: spinal cord, oral cavity (whole), parotid glands, submandibular glands, constrictor muscles (inferior, middle and superior) for all patients, while brain stem, larynx, eyes, chiasm, thyroid, cochleae only when located near to the PTV.

For the parotids the mean dose was constrained to 26Gy to the full gland whenever possible according to eventual overlap with target (a tolerance dose of ~25Gy is reported for long-term salivary function <25 % of baseline [10]).

Near-to-maximum dose to the spinal cord (D_0.1cm3_) was to be kept below 45Gy, while to the brain stem below 54Gy.

The dose to oral cavity should be kept low where possible, maximally avoiding hot spots. The oral cavity delineation included the entire region delimited anteriorly and laterally by the mandible and the gums/dental arches, posteriorly by the pharyngeal posterior wall, superiorly by the hard palate. It was therefore not possible to correlate the high dose point with the mucositis, as suggested by Narayan et al. [9], since it is not possible to correlate it by position, and most of the oral cavity delineated volume is not accessible during clinical inspection. For those reasons, the volume receiving medium-high doses was recorded and analysed instead of the maximum point dose.

Dysphagia threshold dose values to constrictors were found by Levendag et al. [11] to be mean doses of 51, 48 and 32Gy respectively to the superior, middle and inferior constrictors for late toxicity. In this study constrictor muscles and submandibular salivary glands were not included in the optimization process but were considered for dose records. The rationale for this choice was to minimize the risk of geographical miss.

Larynx doses were not evaluated for larynx tumours, as the structure was part of the PTV. Mean laryngeal doses should be kept below the 43.5Gy, indicated as predictive parameter for laryngeal oedema [12, 13]. Thyroid irradiation during HNC treatment could lead to late hypothyroidism (in about 30 % of the patients [14, 15]), for a thyroid irradiation of about 50Gy.

Many quantitative parameters from DVH of all the structures were analysed. For OARs, the dose received by 1/3, 1/2, 2/3 of the structure volume (D_x_), the mean dose, the volume receiving at least 10, 20, 30, 40, 50, 60 and 70Gy were analysed (V_xGy_). For targets, the near-to-minimum and near-to-maximum doses D_98%_ and D_2%_, the volume receiving 95 and 107 % of the prescribed doses were evaluated, together with a conformity index (ratio between the volume receiving 90 % of the prescription dose and the volume of the PTV) and a homogeneity index (ratio between D_5%_-D_95%_ and the mean dose).

### Toxicity assessment

All patients had weekly clinical evaluation during the treatment, and 1 month after the treatment. Patient follow-up continued according to the schedule: every 3 months for the first 3 years, every 6 months for years 4th and 5th and then yearly.

Toxicity was graded according to the Common Toxicity Criteria CTCAE version three, evaluating the oral mucositis, xerostomia, altered taste, dysphagia, dermatitis. Toxicity was defined as acute when occurring during radiotherapy and in the first 3 months after. Late toxicity, evaluated at 6 and 12 months after the end of the radiotherapy treatment, was recorded as well as the outcome.

No patient required percutaneous endoscopic gastrostomy for feeding due to treatment toxicity.

### Toxicity related to dosimetric data, and clinical evaluation

Correlation was explored for OAR dose parameters and related acute and late toxicities. In particular: the oral cavity for the mucosal/taste toxicity, the salivary glands for the salivary toxicity, the constrictor muscles for the dysphagia.

A univariate ANOVA analysis was used considering 0.05 the level of significance, after having confirmed the normal distributions. To account for the multiple comparisons problem the Bonferroni test was applied to the ANOVA analysis.

Preliminary overall and disease free survival were evaluated with Kaplan-Meier analysis.

## Results

### Patient characteristics

Patient characteristics are reported in Table 1.

**Table 1:** Patient characteristics

Number of patients		102
Age [y.o.]	Median [range]	63 [31, 95]
	Mean ± SD	64 ± 12
Gender	Male	72 (71 %)
	Female	30 (29 %)
Tumour site	Larynx	11 (11 %)
	Hypopharynx	13 (13 %)
	Oral Cavity	4 (4 %)
	Oropharynx	48 (47 %)
	Nasopharynx	22 (22 %)
	Nasal/paranasal sinus	4 (4 %)
T stage	1	11 (11 %)
	2	27 (26 %)
	3	25 (25 %)
	4	29 (28 %)
	4a	9 (9 %)
	4b	1 (%)
N stage	0	15 (15 %)
	1	13 (13 %)
	2	14 (14 %)
	2a	4 (4 %)
	2b	32 (31 %)
	2c	18 (18 %)
	3	5 (5 %)
	3b	1 (1 %)
TNM stage	Stage 3	20 (20 %)
	Stage 4a	56 (55 %)
	Stage 4b	26 (25 %)
Histology	Squamocellular carcinoma	89 (87 %)
	Undifferentiated carcinoma	12 (12 %)
	Mucoepidermoid carcinoma	1 (1 %)
Performance Status	PS = 0	69 (68 %)
	PS = 1	24 (23 %)
	PS = 2	9 (9 %)
Induction Chemotherapy	No	47 (46 %)
	TPF	47 (46 %)
	CBCDA + 5FU	2 (2 %)
	CBCDA + adriamicin	1 (1 %)
	CDDP + 5FU	4 (4 %)
	CDDP + adriamicin	1 (1 %)
Concomitant Chemotherapy	No	9 (9 %)
	Cetuximab	19 (19 %)
	Weekly Cisplatinum	64 (63 %)
	Three-weekly Cisplatinum	10 (10 %)

### Dosimetric results

**Fig. 1:**
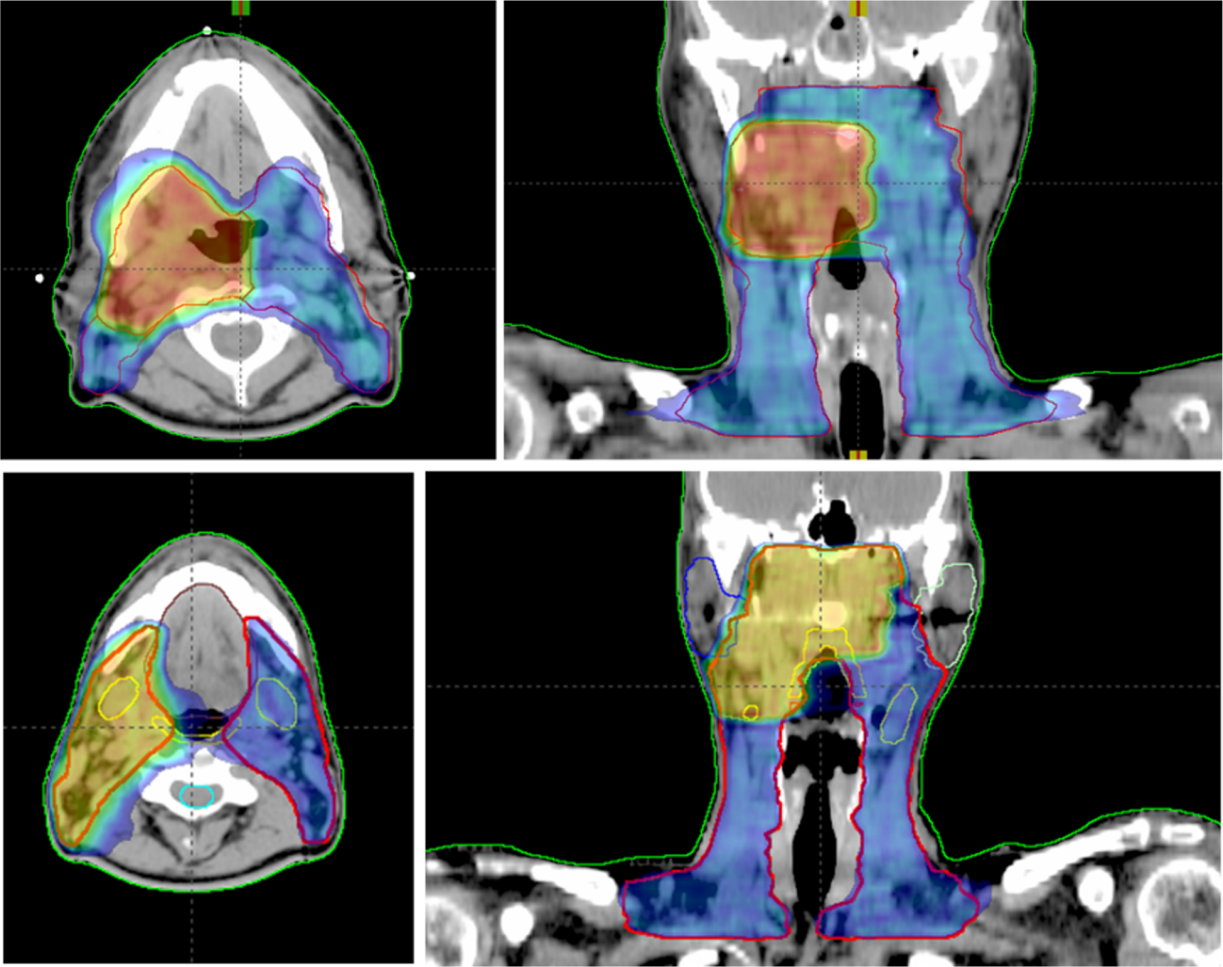
Examples of dose distributions

Dosimetric results are reported in Table 2, where the mean ± StandardDeviation are reported for some parameters for the whole cohort of patients, and stratified per anatomical regions: larynx/hypopharynx, oropharynx/oral cavity, and nasopharynx. Treatment plans fulfilled the dosimetric criteria of target coverage and OAR sparing in almost all the cases.Typical dose distributions are presented in Fig. 1.

**Table 2:** Overall dosimetric parameters (Mean ± StandardDeviation)

Structure	Parameter	All cases	Larynx	Oropharynx	Nasopharynx
			Hypopharynx	Oral cavity	
PTV_69.96Gy	Volume [cm^3^]	263 ± 147	233 ± 168	273 ± 141	273 ± 131
	Mean [Gy]	69.96	69.96	69.96	69.96
	D_2%_ [Gy]	72.5 ± 0.8	72.4 ± 0.7	72.5 ± 0.8	72.8 ± 0.9
	D_98%_ [Gy]	65.3 ± 1.3	65.7 ± 1.0	65.3 ± 1.4	65.2 ± 1.0
	Std.Dev. [Gy]	1.7 ± 0.4	1.6 ± 0.3	1.7 ± 0.4	1.9 ± 0.5
PTV_54.45Gy^a^	Volume [cm^3^]	442 ± 198	459 ± 195	413 ± 181	477 ± 222
	Mean [Gy]	56.2 ± 2.0	56.2 ± 1.6	56.3 ± 2.1	56.2 ± 2.1
	D_98%_ [Gy]	50.9 ± 1.2	50.9 ± 1.1	51.0 ± 1.0	50.9 ± 1.6
	Std.Dev. [Gy]	2.8 ± 1.0	2.9 ± 0.9	2.8 ± 0.9	2.8 ± 1.0
Spinal Cord	D_2%_ [Gy]	36.2 ± 8.1	36.5 ± 5.4	34.6 ± 6.4	38.9 ± 11.5
Brain Stem	D_2%_ [Gy]	34.3 ± 15.9	27.4 ± 12.9	29.1 ± 14.6	47.7 ± 11.3
Constrictor inf.	Mean [Gy]	48.6 ± 13.8	66.6 ± 4.9	44.3 ± 9.4	39.5 ± 10.6
Constrictor mid.	Mean [Gy]	59.2 ± 11.0	67.5 ± 5.1	58.8 ± 9.5	52.4 ± 12.3
Constrictor sup.	Mean [Gy]	62.9 ± 8.8	57.0 ± 9.1	66.1 ± 4.5	62.4 ± 11.1
Oral Cavity	Mean [Gy]	44.9 ± 8.2	37.4 ± 6.5	49.9 ± 6.2	43.0 ± 6.4
	V_50Gy_ [%]	40.5 ± 18.7	23.7 ± 12.5	52.6 ± 15.5	34.6 ± 12.8
Parotids	Mean [Gy]	26.0 ± 7.2	22.5 ± 3.8	27.4 ± 8.0	26.6 ± 7.0
Submandibulars	Mean [Gy]	61.1 ± 10.0	59.2 ± 8.0	63.2 ± 8.1	59.3 ± 13.3
Larynx	Mean [Gy]	38.7 ± 9.0	51.6 ± 13.6^b^	36.9 ± 7.5	38.1 ± 5.8
Eyes	Mean [Gy]		1.3 ± 0.2	1.2 ± 0.4	6.1 ± 6.8
Thyroid	Mean [Gy]	43.9 ± 8.2	47.4 ± 9.4	43.0 ± 7.8	42.5 ± 6.7

^a^subtracting the PTV_69.96Gy volume

^b^only hypopharyngeal tumours

In average, over all the patients, the parotid glands received less than the tolerance of 26 Gy mean dose. Delivered doses were higher for oro-and naso-pharyngeal sites, as expected.

The oral cavity doses were obviously higher for oropharyngeal/oral cavity tumours.

No constraints were used during RT planning in order to reduce the constrictor muscles dose. The middle constrictors received doses substantially higher than the threshold for larynx/hypopharynx tumour locations, while the superior constrictors received in average, on all sites, higher doses than the threshold value (mean dose of 63Gy to compare with the 32Gy threshold).

Mean laryngeal doses were kept, except for hypopharyngeal tumours, in average below the threshold value of 43.5Gy. The mean dose to the thyroid was reported, being higher than 40Gy.

### Toxicity profile

Results for acute and late toxicities are reported in Table 3.

**Table 3:** Toxicity profile

	Acute toxicity		Late toxicity	
	Grade	Nb. patients (%)	Grade	Nb. patients (%)
Mucosal Toxicity	0	15 (15 %)	0	99 (97 %)
	1	33 (32 %)	1	2 (2 %)
	2	43 (42 %)	2	1 (1 %)
	3	11 (11 %)	3	-
Salivary Toxicity	0	74 (73 %)	0	64 (63 %)
	1	24 (23 %)	1	19 (19 %)
	2	4 (4 %)	2	18 (18 %)
	3	-	3	1 (1 %)
Taste Toxicity	0	65 (64 %)	0	82 (80 %)
	1	29 (28 %)	1	13 (13 %)
	2	8 (8 %)	2	7 (7 %)
Swallowing Toxicity	0	49 (48 %)	0	99 (97 %)
	1	21 (20 %)	1	2 (2 %)
	2	26 (25 %)	2	1 (1 %)
	3	6 (6 %)	3	-
Skin Toxicity	0	26 (25 %)	0	102 (100 %)
	1	39 (38 %)	1	-
	2	33 (31 %)	2	-
	3	4 (4 %)	3	-

Acute mucosal and swallowing toxicities higher than grade 3 were reported by only 11 and 6 % of the patients, respectively; late morbidities (G1 and G2, no G3) were present only in 3 % of the cases. Conversely, late salivary toxicity profile was worse than acute toxicity, with 19 % of persisting late grade equal or higher than two. The acute skin toxicity (up to G3 in 4 % of the patients) was totally recovered by all patients.

Another acute morbidity recorded and not reported in the table was acute dysphonia in three patients (all with laryngeal tumours). Other late toxicities were: four patients with hearing loss (mean doses to cochleae in those patients varied from 2 to 66Gy); one patient with laryngeal chondritis (in a laryngeal tumour, with larynx receiving the full 70Gy dose); one patient presented late trismus (part of the temporo-mandibular joint received the full dose of 70Gy).

OAR dose parameters were evaluated to check for possible correlations with the toxicity profile; in Table 4 the mean doses and the significantly correlating dosimetric parameters are reported stratified to the toxicity grade.

**Table 4:** Dosimetric parameters (Mean ± Std. Error of Mean) stratified to the toxicity grading

MUCOSAL TOXICITY		G0	G1	G2	G3
Oral Cavity	V_30Gy_ [%]^*^	73.3 ± 4.4	73.4 ± 2.8	83.2 ± 1.7	84.3 ± 6.9
	V_40Gy_ [%]^*^	53.6 ± 5.2	53.0 ± 3.5	62.3 ± 2.5	70.5 ± 7.8
	V_70Gy_ [%]^*^	6.9 ± 2.8	8.8 ± 1.6	14.9 ± 2.1	17.4 ± 6.6
	Mean [Gy]	42.5 ± 2.7	43.9 ± 1.4	47.7 ± 1.1	49.6 ± 4.4
SALIVARY TOXICITY		G0	G1	G2	G3
Parotid Ipsilateral	Mean [Gy]	30.0 ± 1.2	27.0 ± 1.1	34.9 ± 7.5	
Parotid Contralateral	Mean [Gy]	22.4 ± 0.7	22.9 ± 0.6	23.8 ± 0.3	
Parotids	Mean [Gy]	25.9 ± 0.7	25.1 ± 0.7	29.4 ± 3.7	
Submandibulary Right	Mean [Gy]	61.0 ± 1.3	60.8 ± 1.5	60.5 ± 3.4	
Submandibulary Left	Mean [Gy]	60.9 ± 1.3	62.2 ± 1.2	60.8 ± 3.7	
SWALLOWING TOXICITY		G0	G1	G2	G3
Inferior Constrictor	D_1/3V_ [Gy]^*^	45.9 ± 2.1	55.3 ± 2.6	54.5 ± 2.3	58.8 ± 6.1
	D_1/2V_ [Gy]^*^	43.0 ± 2.1	53.6 ± 2.8	52.0 ± 2.4	56.9 ± 6.4
	D_2/3V_ [Gy]^*^	40.1 ± 2.1	51.4 ± 2.8	49.2 ± 2.5	55.5 ± 6.7
	V_30Gy_ [%]^*^	84.0 ± 3.9	98.9 ± 0.6	98.0 ± 1.1	100.0 ± 0.1
	V_40Gy_ [%]^*^	55.6 ± 5.4	84.4 ± 6.1	78.2 ± 5.3	84.4 ± 13.9
	V_50Gy_ [%]^*^	28.4 ± 5.0	54.8 ± 9.0	52.0 ± 8.1	62.3 ± 18.5
	Mean [Gy]^*^	43.4 ± 2.0	53.5 ± 2.6	51.9 ± 2.3	57.5 ± 6.1
Middle Constrictor	Mean [Gy]	56.8 ± 1.8	59.9 ± 1.9	62.5 ± 1.5	63.1 ± 3.9
Superior Constrictor	Mean [Gy]	62.4 ± 1.5	62.7 ± 1.5	63.6 ± 1.6	60.6 ± 3.1

^*^Significant with *p* < 0.05

#### Acute toxicity

The V_30Gy_, V_40Gy_, and V_70Gy_ dosimetric parameters of oral cavity were found to correlate (univariate ANOVA analysis) with mucosal toxicity grading with p values of 0.01, 0.03, and 0.05, respectively. Similar dose values were found for the G0-1 toxicities, while increasing sensibly for G2 and G3.

Concerning the salivary glands, stratifying according to ipsilateral (relative to the tumour or positive lymphnode side), and no parameters were found significant for toxicity prediction.

Regarding the constrictors and the swallowing toxicity, most of the dosimetric parameters of the inferior constrictor muscle (mean dose, D_1/2V_, D_1/3V_, D_2/3V_) were significant according to the univariate ANOVA analysis and the Bonferroni post-hoc test. The dose values increased with toxicity grading, from G0, G1-2, to G3. No correlations were found for the middle and superior constrictors.

#### Late toxicity

No significant parameters correlated the late mucosal toxicity. The parotid gland volume (all parotids) showed a tendency to be significant, with *p* = 0.055 (*p* = 0.03 for the ipsilateral parotid); the single case of late G3 salivary toxicity presented parotid volumes of 33.8 and 30.3 cm^3^, ipsi-and contralateral respectively, while the average gland volume over all the other patients was 21.6 ± 8.0 cm^3^. No correlations were found for swallowing toxicity and the constrictor muscles dosimetric parameters.

### Clinical outcome

**Fig. 2:**
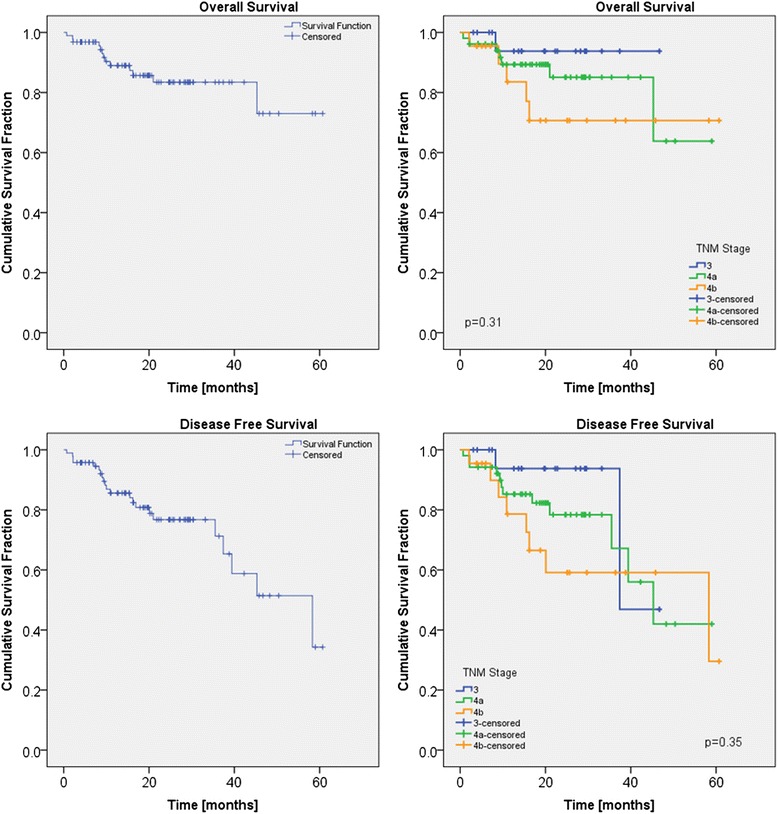
Kaplan-Meier for overall and disease free survivals

Of the 102 patients, 94 were evaluated for overall survival and disease free survival (eight patients were lost to follow-up). Median follow-up time was 19 months (range 1–61 months). Kaplan-Meier survival plots are presented in Fig. 2. Overall Survival figures for the patient cohort are at 3 and 5 years: 83 % ± 4 %, and 73 % ± 10 %. Estimated mean Overall Survival is 51 ± 3 months (46–56 months at 95 % confidence level). Disease Free survival at 2, 3, 4 and 5 years are: 77 % ± 5 %, 71 % ± 7 %, 51 % ± 11 % and 34 % ± 16 %, respectively. Differences among the TNM staging were not significant (in Fig. 2, p > 0.3). Estimated mean Disease Free Survival is 43 ± 3 months (38–49 months at 95 % confidence level).

Sixty-seven patients had complete clinical and radiological response at the first post-treatment visit and imaging; of this group, three patients presented later a loco-regional relapse and are still alive at the moment of the analysis, and one patient died for appearance of distant metastases (lung). Fifteen cases presented a partial response to the treatment with persistence of disease in either primary or nodal sites.

## Discussion

In patients affected by HNC, the predominant pattern of failure is represented by loco-regional relapse. The introduction of IMRT has led to more conformed high-dose regions with better sparing of the OARs. It also allows the delivery of SIB, determining a shorter overall treatment time, hypo-fractionation and dose escalation.

Several studies, randomized and non-randomized, demonstrated a non-inferiority of IMRT compared to 3DCRT in terms of local control [16–19]. Moreover, side effects, such as xerostomia, are proven to be significantly reduced in patients treated with IMRT, even months after the treatment [16].

Different planning studies comparing IMRT with VMAT have been published in the recent years. On average, VMAT plans achieve the same target coverage as IMRT, especially using multiple arcs [4], while slightly better OARs sparing. For example in the Johnston et al. work [18], contralateral parotid sparing was improved with VMAT (mean dose of 25.08Gy vs. 26.18Gy for oropharynx, 31.37Gy vs. 36.59Gy for nasopharynx). On the other hand, there is a lack of consensus for dose prescription in HNC, and significant variations in delivery among centres exist.

The study of Smet et al. [8] evaluated clinical results by comparing two groups of 78 and 79 patients treated with IMRT or RapidArc technique, respectively. The fractionation there used was: 20 fractions of 2 Gy followed by four fractions of 1.6Gy twice a day to the elective volume, followed by 16 fractions of 1.6Gy twice a day to the boost volume (no SIB), for a total treatment time of 6 weeks (30 days). The reported G3 acute mucositis was in 49 % of the patients, G3 dysphagia in 63 % of the patients, and one G4 dysphagia. No doses were reported to neither oral cavity nor constrictor muscles. This toxicity profile is worse than what here reported (11 % G3 mucosal toxicity and 6 % G3 dysphagia, no G4). The main, important, difference between the two studies is the fractionation scheme. Slightly better in the present study are also the overall survival at 3 years (83 % with respect to the 71 % of Smet et al., for similar median follow-up) and the disease free survival at 3 years (71 % with respect to 64 %). However, in both studies a higher number of patients would be needed to evaluate survival differences as significant.

A fractionation schedule more similar to the present study was applied in the work of Doornaert et al. [7], with a SIB of 35 fractions of 1.65 and 2.0Gy each. They reported, using different grading system criteria (RTOG), 49 % of G3 mucositis, 86 % G2 xerostomia, 29 % G3 skin toxicity. Such values are higher than what found in the current study. This is remarkable considering that we prescribed a higher biologically equivalent dose in a smaller number of fractions.

We observed less G3 acute toxicity compared to both the previous two studies, in particular lower was the incidence of G3 skin reactions (4 %) but also the rate of G1 and G2 skin toxicity (38 and 31 %, respectively) compared to the study of Smet et al.

When evaluating patients presenting dysphagia, significant correlation between dosimetric parameters and toxicity was found only for inferior constrictor muscle. Numerous studies identified structures involved in swallowing toxicity [20–27]; dose to the inferior constrictor of the pharynx has been showed to affect the grade of dysphagia in patients treated with IMRT technique in different studies, in particular the mean dose according to Caglar et al. [21], but also V_60Gy_ > 30 % and V_65Gy_ > 60 % for Li et al. [22]. Dose to inferior constrictor (V_40Gy_ to V_65Gy_) was moreover significantly influencing the necessity of PEG tube at 1 year [22].

In our analysis, all the evaluated dosimetric parameters on inferior constrictor muscle only resulted to be significantly influencing the grade of toxicity, in contrast with other published trials. Levendag et al. [11] reported a significant correlation in a univariate analysis between the mean dose of superior and middle (not inferior) constrictors, and severe dysphagia. Christianen et al. [24] demonstrated that mean dose to superior and middle muscles were significantly associated with swallowing dysfunction, while Deantonio et al. [25] confirmed that mean dose to the superior and the middle constrictors >50Gy correlates with G2–3 dysphagia. These differences could be related to the smaller number of patients included in those studies and a less variability of dose to the upper constrictors that usually are close to (sometimes included in) the high-dose volume, rather than the technique used to administer the treatment.

Oral mucositis, in different degree, is a symptom experienced by almost all the HNC patients treated with radiotherapy. Affecting the quality of life of the patients, this toxicity is one of the most limiting the dose escalation trials. To reduce the incidence of mucositis, the oral cavity dose should be limited; IMRT has the ability to better spare mucosa as demonstrated by Sanguineti et al. [28]. Narayan et al. [29] demonstrated that point doses up to 32Gy to the oral cavity are associated with limited mucosal toxicity. In the present study 54 % of patients presented ≥ G2 mucositis, much lower than others reported in literature varying from 73 to 100 % [30, 31]. Our results are difficult to compare with other experiences, because the delineation of oral cavity is not well standardized. However, our inferior rate of toxicity could be even correlated with the use of the SIB and a moderate hypofractionation.

The present study presents some limitations. The main one is the short follow-up, with a median of 19 months. This fact points to the need to continue the study, collecting data for longer follow up, in order to assess definitive late toxicity and outcome. For that reason the study continues, with the aim to report in a next future definitive data with a follow-up of at least 2 years. In addition, the heterogeneity of the site of primary tumour and the rather small number of patients are other limitations of the present study.

## Conclusion

The use of RapidArc technology to improve the OAR sparing associated with a SIB of 1.65 and 2.12Gy/fraction in 33 fractions showed a good toxicity profile and an encouraging trend for overall survival and disease free survival for patients with advanced stage III-IV HNC.

## Ethics, consent and permissions

All patients signed, at hospital admission, consent for the use of their data for retrospective and scientific investigation.
